# Reduction of Dislocated Shoulder of Six Weeks’ Standing

**Published:** 1859-08

**Authors:** Ephraim Ingals

**Affiliations:** Prof. of Mat. Med. in Rush Med. College


ARTICLE II.

### REDUCTION OF DISLOCATED SHOULDER OF SIX WEEKS' STANDING.

BY EPHRAIM INGALS, M.D., PROF. OF MAT. MED. IN RUSH MED. COLLEGE.

This case occurred in a laboring man, aged about fifty years, having a muscular system of medium development, who, six weeks before I saw him, had a dislocation of the shoulder, which had remained unreduced. The humerus was thrown downwards and forwards, its head resting on the pectoral muscles. There was total inability to raise the arm; indeed, motion of the limb in any direction was extremely limited and caused much pain. Though impressed with the dangers that might attend the operation, nevertheless, the deformity was so great, and the inconvenience so serious to the patient, that I determined to attempt the reduction of the bone by the application of such force as I thought justifiable. The necessary preparations having been made for the application of the pulleys, the patient was placed very completely under the influence of a mixture of ether and chloroform, and a suitable degree of extension having been made and kept up for about five minutes, the heel was placed in the axilla. Extension was made by the hand at the same time that that by the pulleys was kept up, the arm being then carried across the body, the foot acting as a fulcrum, the head of the bone was thrown into its natural position, the breaking up of the adhesions being audible to all present. After removing the means by which extension had been made, the elbow was confined to the side, but in a little time the head of the bone seemed to slip from the glenoid cavity, though not nearly so great an extent as at first. Anæsthetics being again used, and Jarvis' adjuster applied, the head of the bone was returned to its proper position, after which a light compress was placed in the axilla, the elbow was carried upwards and bandaged to the chest, and the patient was sent to his home, twenty miles from the city. He returned to me in two weeks.

At this time the head of the bone was perfectly in its place, the appearance of the shoulder being not unlike that of the other, except in a little lack of rotundity, which was not greater than might be expected from atrophy of the deltoid muscle, induced by absolute inaction for a period of two months. At this time I directed that passive motion of the joint be commenced, and the application of embrocations with friction; and if there has been no injury of the nerves of the part to occasion muscular paralysis (which may happen even when the dislocation is immediately reduced), I shall expect nearly a complete restoration of the usefulness of the joint. In the reduction of such dislocations, I think the pulleys better than the adjuster, as they leave the axilla unoccupied, and we are thus enabled to apply power more judiciously to the head of the bone. The length of time after such an injury, when it is justifiable to apply such force as would warrant any hope of reduction, is not very well fixed by practical surgeons; indeed, it must vary somewhat according to the condition of the different cases that present themselves for treatment. For this reason I think it well to record and preserve a clear history of every case in which the reduction of an old dislocation is attempted. The accumulation of such facts will enable the surgeon better to decide whether or not the reduction of such dislocations should be attempted, and, if generally successful or harmless, will serve as his justification should the attempt prove unfortunate in its results, which we should not forget it is always liable to do. I have not collated the general results of this operation, though we know it is often followed by a lack of success, and occasionally by even fatal results; in fact, it is likely to be so oftener than we should be led to believe from the perusal of recorded cases, for we well know that surgeons are more prone to publish the issue of favorable than unfortunate attempts. But when we reflect upon our inability to determine whether or not adhesions have formed between the head of the bone and the axillary artery, and the frightful results that might follow the laceration or injury of that vessel, I think every surgeon should ponder well on all such cases, and attempt their reduction with extreme reluctance.

